# Volume of the thalamus and hypothalamus in the Ts1Rhr and Ms1Rhr mouse models relevant to Down syndrome

**DOI:** 10.17912/micropub.biology.000981

**Published:** 2023-10-30

**Authors:** Jocelyn Arthun, Sebastian Aldaz, Pete Hayes, John S. Roberts, Lisa E. Olson

**Affiliations:** 1 Biology, University of Redlands, Redlands, CA USA

## Abstract

A variety of mouse models for Down syndrome (Trisomy 21) have been created to test hypotheses about the correlation of phenotypes to gene content and copy number. Ts1Rhr mice are trisomic for a region on mouse chromosome 16 that is homologous to 5.3 Mb of human chromosome 21. Ms1Rhr mice are monosomic for this region. Magnetic Resonance Imaging (MRI) has revealed characteristic volumetric changes in the brains of humans with Down syndrome such as reductions in the cerebellum, hippocampus, and brain stem, and increases in the ventricles and thalamus. We used MRI with region of interest analysis to measure the volume of the thalamus and hypothalamus in Ts1Rhr, Ms1Rhr, and euploid control mice (n = 10-11 per group). Ts1Rhr mice had a 6.6% reduction and Ms1Rhr mice had an 8.2% reduction in the volume of the thalamus. Ts1Rhr and Ms1Rhr hypothalamic volumes were equivalent to controls. Conflicting data in mouse models show a lack of clarity on causative roles of regions homologous to human chromosome 21 in phenotypes related to the thalamus and hypothalamus in Down syndrome.

**Figure 1.**
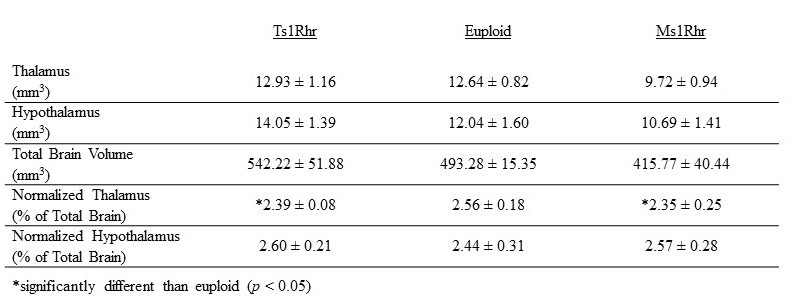
Volume *±* SD of the thalamus and hypothalamus in Ts1Rhr and Ms1Rhr mice compared to euploid controls

## Description

Magnetic Resonance Imaging (MRI) has been used to evaluate abnormalities in brain development in Trisomy 21/Down syndrome (Baburamani, Patkee, Arichi, & Rutherford, 2019) . MRIs of humans with Down syndrome have shown that they have smaller overall brain volume and specific reductions in the cerebellum, brain stem, and hippocampus (Aylward et al., 1999; Pinter, Brown, et al., 2001; Pinter, Eliez, Schmitt, Capone, & Reiss, 2001; Raz et al., 1995; White, Alkire, & Haier, 2003) . Individuals with Down syndrome have enlarged ventricles (Pearlson et al., 1998; White et al., 2003) and a recent preprint reveals that human Down syndrome neonates have enlarged thalami normalized to total brain volume (Fukami - Gartner et al., 2022) .

A variety of mouse models for Down syndrome have been created to test hypotheses about the correlation of phenotypes to gene content and copy number and to test possible interventions (Baburamani et al., 2019; Stagni & Bartesaghi, 2022) . Ts1Rhr mice (Olson, Richtsmeier, Leszl, & Reeves, 2004) are segmentally trisomic for a region on mouse chromosome 16 that is homologous to human chromosome 21, bordered by the *Cbr1* (ENST00000290349.11) and *Mx2* (ENST00000330714.8) genes. This region containing 30 protein-coding genes is 5.3 Mb in humans and 3.9 Mb in mice. Ms1Rhr mice are monosomic for this region (Olson, Richtsmeier et al., 2004) .

To elucidate the mechanism of various brain phenotypes in Down syndrome, mouse models such as these have been heavily investigated by MRI. The array of DS mouse models have various overlapping segments of trisomy, allowing cross-referencing between gene content/copy number with phenotype severity. For example, the Ts65Dn, Ts1Cje, Ts1Rhr, Tc1, Ts66 Yah, and Dp1Yey models all have enlarged ventricles according to volumetric MRI (Duchon et al., 2022; Duchon et al., 2021; Powell et al., 2016; Raveau et al., 2017; Roubertoux et al., 2017) . Ts65Dn, Ts1Cje, Ts1Rhr, and Tc1 mice have reduced cerebellar volumes to various extents (Aldridge, Reeves, Olson, & Richtsmeier, 2007; Baxter, Moran, Richtsmeier, Troncoso, & Reeves, 2000; Olson, Roper, et al., 2004; Olson et al., 2007; Powell et al., 2016) . Although they have hippocampal dysfunction (Belichenko et al., 2009) , the volume of the hippocampus measured by MRI is unchanged in Ts65Dn and Ts1Rhr mice (Olson et al., 2007) ; the hippocampus is larger in Tc1 mice (Powell et al., 2016) .

The Ms1Rhr mouse model, unlike those listed above, is a model for monosomy of genes in the proposed critical region. Ms1Rhr mice have an enlarged cerebellar volume but reduced hippocampal volume when normalized to total brain volume (Aldridge et al., 2007; Olson et al., 2007) . Phenotypes for monosomy and trisomy are not always in opposite directions. Structures that are influenced in the same way by trisomy and monosomy may have developmental pathways that are similarly perturbed by either over- or under-expression of the same genes. Thus, the evaluation of monosomic mouse models can be exploratory in nature.

The thalamus (integrator of sensory information to the cortex) and hypothalamus (homeostasis and hormonal control) regions have not been as intensely studied in mouse models of Down syndrome, but some work has been done in this area. One MRI study of a mouse model transgenic for a relatively small region (a 570 kb yeast artificial chromosome) showed an increase in the thalamus-hypothalamus volume analyzed together (Sebrie et al., 2008) . The thalamus was enlarged in the Tc1 mouse model, with the hypothalamus equivalent to controls (Powell et al., 2016) . Conversely, the Dp1Yey mouse has a larger hypothalamus and unaltered thalamus (Duchon et al., 2021) . The volume of thalamus and hypothalamus were normal in Ts65Dn, Ts66Yah, and Ts1Rhr (Duchon et al., 2022; Duchon et al., 2021) , but this analysis also was unable to replicate previously shown reductions in cerebellar and hippocampal volume in the Ts65Dn model, calling into question the sensitivity of the method or power due to sample size (n=5-7 per group). Thus, a clear relationship between additional copies of which or how many genes would be necessary and sufficient to cause phenotypes in the thalamus and hypothalamus has not emerged.

Results:

The volumes of the thalamus and hypothalamus were analyzed by drawing regions of interest on each contiguous slice of a high resolution MRI. Because the total volume of the brain is different in Ts1Rhr and Ms1Rhr mice compared to euploid controls (Olson et al., 2007) , we normalized the volumes of the thalamus and hypothalamus to the total brain volume in each mouse. Ts1Rhr mice had a 6.6% reduction and Ms1Rhr mice had an 8.2% reduction in the normalized volume of the thalamus compared to euploid (Table 1, p < 0.05). Ts1Rhr and Ms1Rhr hypothalamic volumes were statistically equivalent to euploid controls.

## Methods

Mice were bred and maintained as previously described and were of a F _1_ (B6·129) background (Olson et al., 2007) . The sample sizes for the thalamus were 10 Ts1Rhr (7 M, 3 F), 10 Ms1Rhr (4 M, 6 F), and 10 euploid (5 M, 5 F); the sample sizes for hypothalamus were 10 Ts1Rhr (7 M, 3 F), 10 Ms1Rhr (4 M, 6 F), and 11 euploid (6 M, 5 F) mice. Mice were examined at 8–9 weeks of age.

MRIs were captured in the Reeves laboratory at The Johns Hopkins School of Medicine via a 400 MHz Omega NMR Spectrophotometer interfaced to a 9.4T/89mm vertical bore magnet as previously described (Baxter et al., 2000; Olson et al., 2007) . The software program MRIcro 1.3 ( https://people.cas.sc.edu/rorden/mricro/mricro.html ) was used to draw regions of interest confirmed in all three planes and calculate volumes (0.000035937 mm ^3^ /voxel).

Landmarks were determined using atlases (braininfo.rprc.washington.edu; Paxinos & Franklin, 2001). Boundaries used for the thalamus were the hippocampus and D3 ventricle, lateral ventricles, and the edge of the mammillothalamic tracts. For the hypothalamus, boundaries were the thalamus, the ventral surface of the brain, the lateral hypothalamic area, rostral to the posterior nucleus of the hypothalamus, and caudal to the subthalamic nucleus. Due to difficulty in distinguishing clear borders, the optic chiasm, nucleus of stria terminalis, ventral tegmental area, mammillothalamic tract, and the fornix were also included in the “hypothalamus region.”

Data were analyzed using SPSS (IBM, Armonk, NY) using an alpha of 0.05. Normalized hypothalamus volume was analyzed with a one-way ANOVA. Due to non-homogeneity of variance, normalized thalamus volume was analyzed with a nonparametric Kruskal-Wallis test.
